# Double-edged sword: Granulocyte colony stimulating factors in cancer patients during the COVID-19 era

**DOI:** 10.6061/clinics/2020/e2033

**Published:** 2020-07-02

**Authors:** Ali Alkan, Asaf Uncu, Irmak Taşkıran, Özgür Tanrıverdi

**Affiliations:** I Medical Oncology, Muğla Sıtkı Koçman University School of Medicine, Muğla, Turkey; II Internal Medicine, Muğla Sıtkı Koçman University School of Medicine, Muğla, Turkey

To the Editor,

The whole world is struggling because of coronavirus disease (COVID-19), and it has already triggered a series of crises. Patients with comorbidities are highly susceptible to infection and thus have a poor prognosis. Cancer patients have been heavily impacted by the onset of this pandemic. A few recent studies on cancer patients have shown that they are at an increased risk for severe acute respiratory syndrome-coronavirus-2 (SARS-CoV-2) infection. Furthermore, infected patients were found to have an increased risk of admission to the intensive care unit, requiring invasive ventilation, or death (1). Oncologists are providing optimal cancer care and, simultaneously, are trying to protect these fragile patients from SARS-CoV-2 infection and its consequences. Cytopenia, especially neutropenia and lymphopenia, is the most frustrating complication of cancer treatment and can increase the susceptibility to infection. Both the European Society for Medical Oncology (ESMO) and the National Comprehensive Cancer Network (NCCN) have recommended expanding the indications for use of granulocyte colony-stimulating factors (G-CSFs) in cancer patients to decrease the risk of neutropenic fever and prevent them from having to visit a hospital (2,3). The efficacy of G-CSFs in preventing febrile neutropenia and treatment-related hospitalizations has been well documented in earlier studies. Moreover, a 5-day course of G-CSF prophylaxis in most chemotherapy regimens has been reported to be effective (4); however, limited data are available on its efficacy in COVID-19 patients.

G-CSFs are growth factors that stimulate the survival, proliferation, differentiation, and function of neutrophil precursors and mature neutrophils via signal transduction pathways. Studies on healthy volunteers revealed that the administration of G-CSFs resulted in increased the levels of interleukin (IL)-1, soluble tumor necrosis factor (TNF) receptors (sTNF-Rs), IL-6, IL-8, and IL-10 and reduced the levels of TNF-α, interferon (IFN)-γ, and granulocyte-macrophage colony-stimulating factor (GM-CSF) (5). Data from a limited number of COVID-19 patients have shown a cytokine storm in critically ill patients. Besides, the levels of IL-2, IL-6, IL-10, and IFN- γ were found to be higher in severe cases of COVID-19 than in mild cases, and a strong inflammatory response during its clinical course was reported to be associated with high morbidity and mortality (6). Clinical outcomes are also unpredictable in COVID-19 patients with high levels of inflammatory cytokines undergoing G-CSF prophylaxis. Furthermore, there are chances of acute respiratory failure during G-CSF-induced neutropenia recovery. G-CSFs have been reported to be associated with activation of the oxidative burst mechanism within circulating or resident alveolar neutrophils and macrophages, further complicating the situation (7,8). Therefore, devastating results can potentially be seen in COVID-19-positive patients administered G-CFSs or in those exposed to infection while undergoing G-CSF therapy. However, very limited data on the outcomes of chemotherapy in cancer patients in the COVID-19 era are currently available, with no specific subgroup analysis in patients treated with G-CSFs.

Although the described scenarios are theoretical and speculative, both NCCN and ESMO have concluded that the benefits of G-CSF administration outweigh its risks and have, therefore, recommended its use with caution. Until more data are available, clinicians should be more cautious with the use of G-CSFs in cancer patients in the COVID-19 era.

## References

[1] Liang W, Guan W, Chen R, Wang W, Li J, Xu K (2020). Cancer patients in SARS-CoV-2 infection: a nationwide analysis in China. Lancet Oncol.

[2] ESMO (2020). SUPPORTIVE CARE STRATEGIES DURING THE COVID-19 PANDEMIC.

[3] NCCN (2020). NCCN Hematopoietic Growth Factors. Short-Term Recommendations Specific to Issues with COVID-19 (SARS-CoV-2).

[4] Clemons M, Fergusson D, Simos D, Mates M, Robinson A, Califaretti N (2020). A multicentre, randomised trial comparing schedules of G-CSF (filgrastim) administration for primary prophylaxis of chemotherapy-induced febrile neutropenia in early stage breast cancer. Ann Oncol.

[5] Hartung T, Docke WD, Gantner F, Krieger G, Sauer A, Stevens P (1995). Effect of granulocyte colony-stimulating factor treatment on ex vivo blood cytokine response in human volunteers. Blood.

[6] Liu J, Li S, Liu J, Liang B, Wang X, Wang H (2020). Longitudinal characteristics of lymphocyte responses and cytokine profiles in the peripheral blood of SARS-CoV-2 infected patients. Version 2. EBioMedicine.

[7] Azoulay E, Darmon M, Delclaux C, Fieux F, Bornstain C, Moreau D (2002). Deterioration of previous acute lung injury during neutropenia recovery. Crit Care Med.

[8] Karlin L, Darmon M, Thiéry G, Ciroldi M, de Miranda S, Lefebvre A (2005). Respiratory status deterioration during G-CSF-induced neutropenia recovery. Bone Marrow Transplant.

